# Semaphorins as emerging clinical biomarkers and therapeutic targets in cancer

**DOI:** 10.7150/thno.54023

**Published:** 2021-01-15

**Authors:** Roberta Mastrantonio, Hua You, Luca Tamagnone

**Affiliations:** 1 Università Cattolica del Sacro Cuore, Rome, Italy.; 2 Affiliated Cancer Hospital & Institute of Guangzhou Medical University, China.; 3 Fondazione Policlinico Universitario “A. Gemelli”, IRCCS, Rome, Italy.

**Keywords:** prognostic biomarkers, predictive biomarkers, therapy, cancer, semaphorin, plexin, neuropilin

## Abstract

Semaphorins are a large family of developmental regulatory signals, characterized by aberrant expression in human cancers. These molecules crucially control cell-cell communication, cell migration, invasion and metastasis, tumor angiogenesis, inflammatory and anti-cancer immune responses. Semaphorins comprise secreted and cell surface-exposed molecules and their receptors are mainly found in the Plexin and Neuropilin families, which are further implicated in a signaling network controlling the tumor microenvironment. Accumulating evidence indicates that semaphorins may be considered as novel clinical biomarkers for cancer, especially for the prediction of patient survival and responsiveness to therapy. Moreover, preclinical experimental studies have demonstrated that targeting semaphorin signaling can interfere with tumor growth and/or metastatic dissemination, suggesting their relevance as novel therapeutic targets in cancer; this has also prompted the development of semaphorin-interfering molecules for application in the clinic. Here we will survey, in diverse human cancers, the current knowledge about the relevance of semaphorin family members, and conceptualize potential lines of future research development in this field.

## Introduction

Semaphorins were initially identified for their function as short-range axonal guidance molecules during neuronal development, but actually they are involved in the regulation of a wide spectrum of developmental processes and human diseases 1. Semaphorins are a family of cell surface exposed and soluble proteins conserved from invertebrates to humans. In vertebrates, 20 semaphorin genes have been identified, which are divided into five classes based on sequence and structure similarities 2. The structural hallmark of all semaphorins is an N'‑terminal 500 amino acid long sema domain, assuming a seven-blade β‑propeller shape. The sema domain is tightly coupled to an adjacent small cysteine-rich domain, called the PSI (plexin-semaphorin-integrin) domain. Beyond the sema and PSI domains, in the extracellular moiety of many semaphorins are found immunoglobulin (Ig)-like domains or thrombospondin type 1 repeats. The main semaphorin receptors belong to the plexin gene family, which includes nine members divided into four subgroups 3. Most secreted class 3 semaphorins, however, are unable to interact with plexins directly, but instead bind to receptor complexes including plexin-associated co-receptors of the neuropilin family (NRP1 and NRP2).

Notably, semaphorins can signal in autocrine, paracrine, and juxtacrine manner. Transmembrane semaphorins are also capable of bi-directional signaling, acting both *forward* through plexins, and in *reverse* mode via their own cytoplasmic domains. In addition to their interaction in *trans*, whereby a semaphorin exposed on the cell surface can bind to receptors expressed on adjacent cells, certain transmembrane semaphorins were also found to associate in *cis* with plexins on the surface of the same cell 4. Intracellular semaphorin signaling is only partly understood. It can control cell-substrate adhesion and cytoskeletal dynamics through monomeric GTPases, as well as impact cell viability and growth via phosphorylation cascades, just to name two examples. Moreover, recent evidence indicates the relevance of semaphorin signals in the regulation of phenotypic plasticity and cell stemness, implicating an impact on gene transcription 5.

Semaphorins have emerged as crucial regulators of tumor growth and metastasis; in particular, they can control cancer development and progression by acting on the cross-talk between different cell types in the tumor microenvironment 6,7. Taking into account this scenario, it is not surprising to find a continuously growing number of studies supporting the identification of semaphorins as cancer biomarkers. Moreover, substantial preclinical data have provided evidence featuring semaphorin signals as attractive therapeutic targets in cancer, and the first clinical studies with anti-semaphorin drugs are emerging. Notably, the state-of-the-art in this field indicates a notable heterogeneity in the relevance and activities of individual semaphorins in diverse tumor types. Thus, in this article, we will also systematically review the literature implicating any semaphorin family members in specific human cancers.

## Semaphorins as clinical biomarkers in human cancers

**Breast Cancer**. Breast cancer is the most common malignancy in women, and one of the three most common cancers worldwide. It consists of at least four different clinically relevant molecular subtypes: luminal A or B, expressing hormonal receptors for estrogen (ER) and/or progesterone (PR); HER2 oncogene-overexpressing; and triple negative/basal like (HER2 negative and ER/PR negative). Multiple semaphorins have been implicated in breast cancer (also see Table 1).

An elevated expression of the secreted semaphorin **Sema3B** in breast cancer samples has been associated with better prognosis. In fact, *SEMA3B* gene is located in chromosome region 3p21.3, a site of frequent allele loss in the early pathogenesis of lung and breast cancers, consistent with a putative tumor suppressor activity for this semaphorin 8. Notably, *SEMA3B* is also a direct target of the transcription factor GATA3, with a fundamental role in maintaining normal mammary gland homeostasis 9. Sema3B binds NRP1 or NRP2 co-receptors, in complex with Plexins of the A subfamily, PlexinA2 in particular 10. Sema3B can downregulate phosphatidylinositol 3-kinase (PI3K)/Akt activity in cancer cells, inhibit cellular proliferation and tumor growth 8, as well as metastasis 9. Apart from its relevance as favorable prognostic indicator for breast cancer, Sema3B is also under consideration for innovative therapeutic approaches, although caveats remain based on a previous report suggesting that its tumor suppressing activity may trigger rebound inflammatory and prometastatic effects 11.

**Figure 1:**
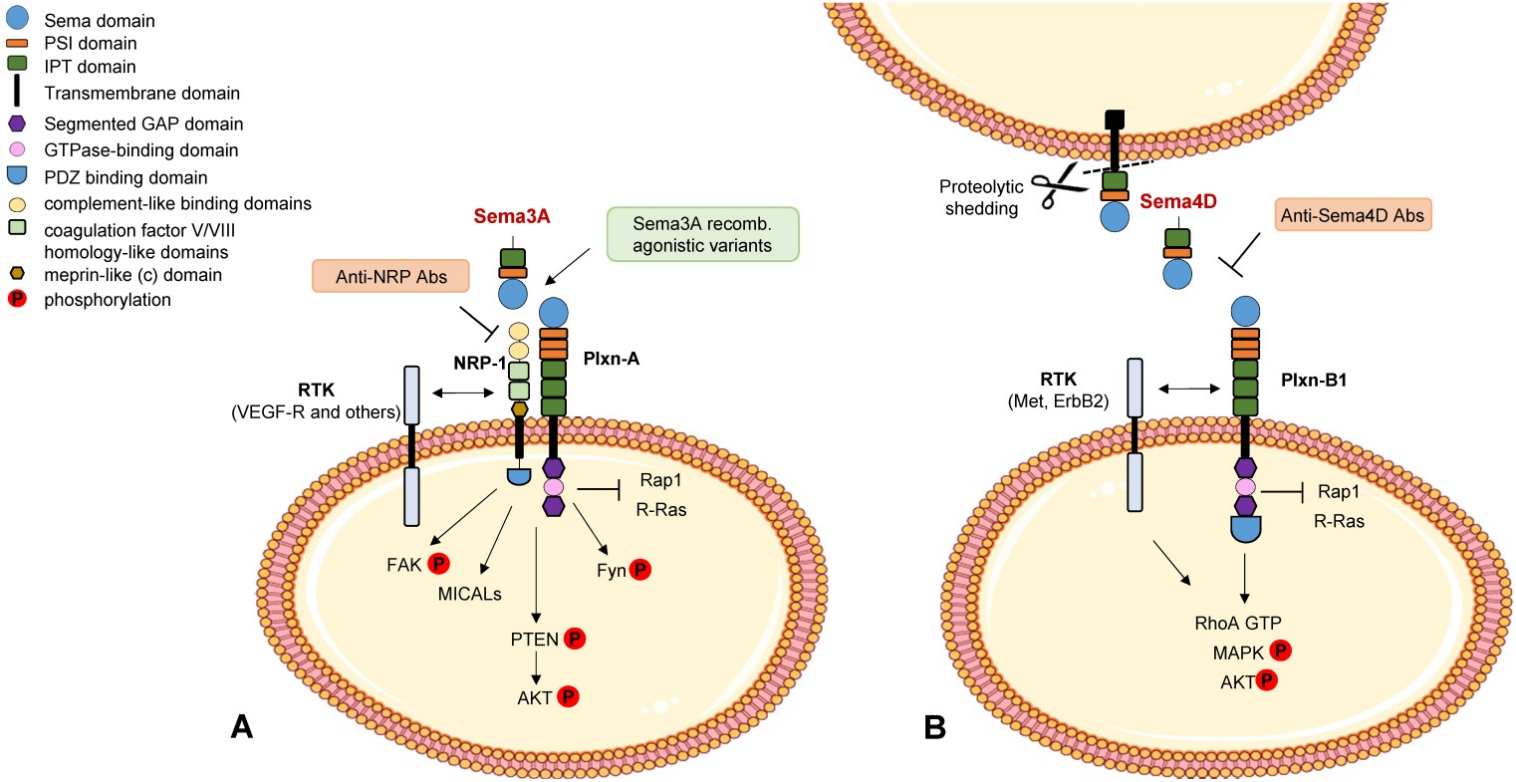
** Semaphorin signaling in cancer cells.** Schematic representation of best known signaling mechanisms mediated in cancer cells by prototypic family members Sema3A (panel A) and Sema4D (panel B) (for a review see 7). The intracellular domain of the plexins deploys GAP activity for Rap1 and R-Ras monomeric GTPases. RTK indicates receptor tyrosine kinases associated with neuropilins and plexins. A few validated molecular tools interfering with these cascades are also indicated (in painted boxes).

High levels of another secreted class 3 semaphorin, **Sema3A**, have also been found to correlate with better patient prognosis, consistent with its ability to induce PTEN activation, through the receptor NRP1, and negatively control breast tumor growth in mouse preclinical models 12 (see Figure 1A). However the functional role of Sema3A/NRP1 signaling in breast cancer cells appears complex, since it was also found to promote stemness and mammosphere formation via MICAL3-dependent stabilization of the regulatory factor Numb 13. Additional studies in other tumor models, discussed below in this review, have further underscored the complexity of Sema3A activities in the tumor microenvironment.

It was reported a higher **Sema3E** expression in breast cancer samples associated with distant metastasis 14. This is consistent with previous experimental findings which indicated a pro-invasive and pro-metastatic activity of this semaphorin in other tumor models 15 via its specific receptor PlexinD1, although the implicated mechanisms remain controversial. Interestingly, a modified recombinant isoform of Sema3E with inhibitor activity has been successfully applied to suppress angiogenesis and tumor progression in mouse models 16. The impact of Sema3E on tumor angiogenesis is consistent with its recognized important role in the regulation of heart and vessels development 17.

**Figure 2:**
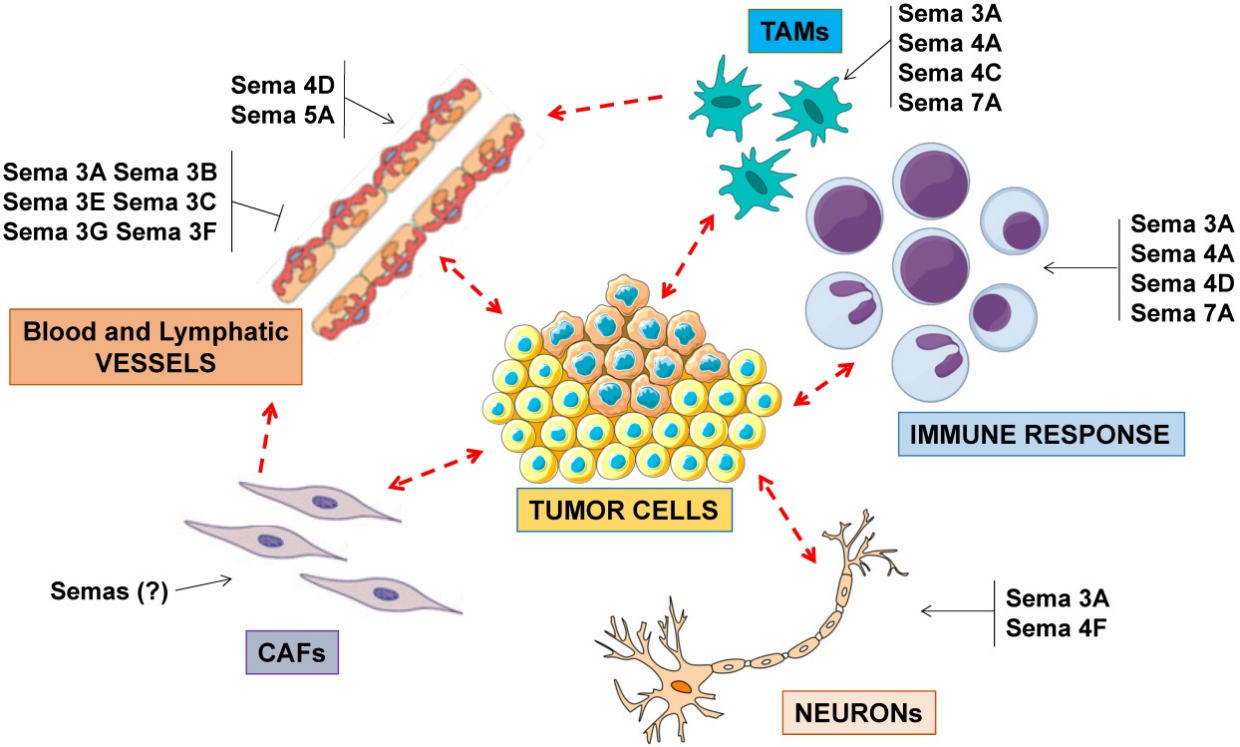
** Semaphorin signaling in the tumor microenvironment.** Semaphorins are major regulators of angiogenesis, acting as inhibitors or as promoters of endothelial cell functions. Other semaphorins regulate the activity of tumor-associated macrophages (TAMs), in turn impacting on immune response and angiogenesis. A subset of so-called immunoregulatory semaphorins have also been implicated in tumor immune-evasion. In addition to their role in neural development, semaphorins may also control the functional coupling between cancer cells and neuronal projections found in the tumor microenvironment.

In general, several semaphorins have been implicated to control cancer progression by impinging on stromal and inflammatory cells present in the tumor microenvironment, beyond cancer cells (Figure 2). A detailed discussion of the known mechanisms of semaphorin-dependent regulation of angiogenesis and immune response can be found in other review articles (e.g. 3,18-22).

Elevated levels of the transmembrane semaphorin **Sema4C** have been correlated with poor outcome of breast cancer. For instance, it was demonstrated that Sema4C forward signaling (via its receptor PlexinB2) is specifically required for breast cancer cell growth 23. In fact, targeting Sema4C/PlexinB2 signaling resulted in cell cycle arrest in G2/M phase, upregulation of tumor-suppressor genes and cell senescence. The underlying mechanism involves the activity of a plexin-associated RhoA exchanger protein, LARG, which maintains elevated RhoA-GTP levels in the cells. Moreover, an elevated Sema4C expression in luminal-type ER+ breast tumor cells was associated with a major phenotypic and behavioral switch, including reduced sensitivity to hormonal therapy and the acquisition of invasive/metastatic properties 23. Notably, increased Sema4C expression in breast cancer cells has also been associated with resistance to paclitaxel-based chemotherapy, while knocking-down its expression could partly recover drug sensitivity 24.

Indeed, metastasis formation is the most important pitfall for breast cancer patients, which otherwise can be cured in a large proportion. Notably, Sema4C was also found to induce ID1 and ID3 transcriptional regulators in triple-negative breast cancer cells, through the enhancement of TGFbeta/BMP signaling; this pathway has been previously implicated in the partial EMT reversion phenotype observed in late stages of the metastatic process 25, which further identifies Sema4C as a relevant driver of breast cancer metastatic progression.

Tumor-associated lymphatic vessels can play a major role in breast cancer metastasis, and it was shown that cancer cell dissemination by this route may be promoted by Sema4C expression in lymphatic endothelial cells (LECs) 26. A subsequent study revealed that Sema4C also fosters cytokine-dependent macrophage recruitment in the TME 27. Notably, soluble Sema4C can be released from the cell surface due to proteolytic cleavage by matrix metalloproteinases (MMPs), and it was proposed as promising serum marker for the early diagnosis and the assessment of metastasis risk in breast cancer 26. Interestingly, two clinical trials aimed at the assessment of these hypotheses have been planned (NCT03663153; NCT03662633).

Another semaphorin consistently implicated in breast cancer development is **Sema7A**. In normal breast tissue Sema7A is almost absent, while its expression increases in breast tumor samples, and it is associated with decreased overall patient survival 28. In fact, Sema7A is considered a tumor promoter, impinging on tumor growth, invasiveness, EMT and metastases, probably by signaling via integrin-β1 receptor 29. Additionally, tumor derived Sema7A can hijack the breast tumor microenvironment by increasing the recruitment of macrophages with pro-angiogenic activity and the growth of tumor-associated lymphatic vessels 28. Recent studies revealed a tumor-promoting role of Sema7A during postpartum mammary gland involution 30,31. Intriguingly, the tissue microenvironment created during this physiological process has proven to be tumor promoting. Actually, Sema7A is highly expressed by mammary epithelial cells during involution; and the study of Sema7A-deficient preclinical mouse models revealed that this semaphorin is crucial to elicit the recruitment of circulating macrophages in the microenvironment, promoting lymphangiogenesis. Moreover, the association of elevated Sema7A and macrophage markers predicted shorter distant metastasis-free survival in a large cohort of breast cancer patients 30. The same group further analyzed the clinical significance of this biomarker in a large panel of ER+ breast cancers, which are often responsive to hormonal deprivation, apart from a fraction that shows primary drug refractoriness or later incurs in drug-resistant relapses. Not only was it confirmed that Sema7A expression is a strong biomarker of shorter patient survival, but it was found in association with late recurrence, correlated with resistance to hormonal therapy. Experimental evidence demonstrated the role of Sema7A signaling in conferring resistance to estrogen deprivation and promoting disease progression and metastasis 32.

Thus, in addition to Sema4C, Sema7A could represent another potential therapeutic target and a predictive biomarker for ER+ breast cancer patients at high risk for resistance to hormonal therapy and metastatic relapse. Notably, previous reports showed that Sema7A is partly shed from the cell surface into the tumor microenvironment and can be detected in the blood, which could enable a convenient assessment of its level by liquid biopsies. A recent study furthermore demonstrated the activity of Sema7A released in association with cancer cell derived exosomes 33.

The role of **Sema4D** in breast cancer appears quite complex based on current literature. Sema4D is a transmembrane protein (capable of short range cell-cell signaling); however, upon shedding by MT1-MMP it is converted into a soluble form, which may bind to its main receptor PlexinB1 expressed on the surface of multiple cell types in the tumor microenvironment, including cancer cells, endothelial cells and immune cells. In fact, Sema4D/PlexinB1 signaling has been reported to promote tumor angiogenesis and invasive growth in experimental models 34,35. Two main downstream mechanisms have been implicated in these functions: the transactivation of tyrosine kinase receptors Met and ErbB2, and the activation of the GTPase RhoA, with consequent phosphorylation of MAPK and Akt effector cascades (see Figure 1B). Moreover, Sema4D was found to mediate RhoA phosphorylation, a regulatory mechanism of invasion and metastasis currently under investigation 36.

In keeping with these data, Sema4D knock-down in breast cancer cells was found to suppress tumor growth *in vivo*, angiogenesis, and bone metastases formation in mouse models 36. At odds with these findings, Sema4D and PlexinB1 levels detectable in human breast tumor samples appear of unclear prognostic significance. For instance, higher expression of Sema4D and PlexinB1 has been statistically associated with ER-positive tumors, characterized by better prognosis 37,38. However, divergent findings were reported in other studies (e.g. 39), suggesting that multivariate analysis of larger case panels is required to elucidate this issue. Moreover, due to their distribution in diverse cell types in the tumor microenvironment, the detection of Sema4D and its receptor might reflect the representation of other components beyond cancer cells.

Interestingly, breast carcinoma cells show high propensity to metastasize to bones, where they usually form osteolytic lesions. In bone homeostasis, Sema4D has an osteoblast-inhibitor activity and it is normally produced by osteoclasts 40; however, in metastatic lesions a locally increased concentration of Sema4D can promote osteolysis. Notably, it was reported that breast cancer patients treated with the anti-hormonal drug tamoxifen have reduced plasmatic levels of Sema4D 41, an effect which could potentially account for some of the reported bone-protective effects of this drug. The same mechanism was not observed in response to another class of hormonal drugs, the aromatase inhibitors (AI), which may further negatively affect bone mass due to deprivation of bone protective estrogens.

Another recent study unveiled that Sema4D expression in primary breast tumor samples is associated with the formation of brain metastasis, consistent with the evidence of Sema4D activity promoting cancer cell extravasation in an experimental model of blood-brain barrier 42. The relevance of Sema4D in the tumor microenvironment was confirmed in multiple tumor types beyond breast carcinoma, which has prompted the preclinical validation of targeted antibodies interfering with its function 43. In particular, the treatment with an anti-Sema4D antibody was found to promote anti-cancer immune response in mouse models by favoring the recruitment of cytotoxic T lymphocytes 44,45. These studies unveiled a further putative function of this semaphorin, fostering immune evasion in the tumor microenvironment. A humanized version of this anti-Sema4D antibody has been validated for application in clinical trials 46 and it is currently under investigation in a phase 1b/2 study, in combination with immune checkpoint inhibitors (NCT03268057). Notably, recent data challenged the view that targeting Sema4D in tumors is always beneficial 47, suggesting that further investigation may be help to exploit Sema4D at its best as therapeutic target in cancer.

**Ovarian cancer.** Ovarian cancer is the leading cause of death among gynecologic cancers. Most symptoms in early stage ovarian cancer are nonspecific, and difficult to diagnose before spreading occurs. A consistent tumor angiogenesis is known to drive the growth and metastatic dissemination of ovarian cancers, leading to poor prognosis. VEGF plays a critical role in vessel formation under both physiological and pathological conditions. Moreover, diverse semaphorin members of the class 4 promote angiogenesis. One important example is **Sema4D**, which mediates endothelial cell chemotaxis, microvessel formation and distant metastasis, via its receptor PlexinB1. Indeed, Sema4D and VEGF levels increase in association with higher malignancy of epithelial ovarian carcinomas. Univariate analysis indicated that the overall survival and progression-free survival of epithelial ovarian cancer patients with high expression of Sema4D were lower than in patients with Sema4D-negative tumors. Together with VEGF, Sema4D is thereby considered an independent adverse prognostic factor for epithelial ovarian cancer patients 48.

**Sema3C** gene was originally identified for being overexpressed in cisplatin-resistant ovarian cancer cells 49, and it was found to be readily induced by diverse chemotherapeutic drugs and by ionizing radiations. Experimental evidence showed that Sema3C expression was responsible for sustaining drug-refractoriness, and capable to raise primary resistance in cisplatin-sensitive cells 49. These findings are furthermore consistent with a recent broad bioinformatic analysis of semaphorin expression data in human tumors, which indicated a significant inverse correlation between Sema3C expression and sensitivity to anti-cancer drugs 50. Notably, NRP1, a known co-receptor of Sema3C, has been repeatedly implicated in cancer cell resistance to therapy 51.

The elevated expression of another secreted semaphorin, **Sema3E**, has been associated with high-grade ovarian endometrioid carcinomas, but not other ovarian epithelial tumors, and found to promote tumor cell invasiveness via its specific receptor PlexinD1 52. It was reported instead that **Sema3A** mRNA and protein expression is lower in ovarian carcinoma compared to normal tissue, and tumors with lowest levels are significantly associated with higher FIGO stage, histological grade, and the presence of lymphatic and distant metastasis 53. Sema3A could therefore represent a valuable prognostic marker for ovarian cancers; moreover, its activity could be potentially exploited for therapeutic approaches, currently validated in other preclinical tumor models in mice 54,55, as discussed later in this review.

**Cervical cancer.** Cervical cancer is the second most common cause of cancer-related death in women worldwide. Infection by specific human papillomavirus (HPV) subtypes and other risk factors have been implicated in the pathogenesis of cervical cancer. Elevated** Sema4C** expression has been associated with poor prognosis in cervical cancer patients. Similar to what reported above for breast cancer, experimental evidence indicated that Sema4C can promote EMT; moreover, it was found to sustain resistance to cisplatin-based chemotherapy in cervical cancer cells, while knocking-down its expression partly recovered drug sensitivity 56.

Moreover,** Sema4D** plays an important role in cervical cancer, due to its involvement in lymphangiogenesis and tumor cell migration 57. Tumors with high expression of Sema4D showed abundant lymphangiogenesis, lymphatic vessel invasion, and the occurrence of lymph node metastases, possibly due to increased VEGF-C/D expression. In addition, analysis of survival data provided compelling evidence that high levels of Sema4D were significantly associated with an unfavorable outcome in cervical cancer patients. PlexinB1 inhibition experiments demonstrated that its ligand Sema4D enhances cervical cancer cell invasive potential. Sema4D⁄PlexinB1 signaling enhanced cell invasiveness through the activation of RhoA, MAPK and AKT pathways. These data suggests an important association between high Sema4D expression and reduced survival of cervical cancer patients 57. Therefore, Sema4D may serve as prognostic biomarker of disease progression, and potentially suggest the adoption of differential protocols for the therapy and patient follow-up.

**Sema5A**, has also been identified as a novel proangiogenic molecule with elevated expression in stage IIb cervical cancer, compared to earlier stages. Sema5A overexpression was significantly associated with lymphangiogenesis, poor prognosis, and the metastatic potential of cervical cancer cells. PlexinB3 is a Sema5A receptor. Importantly, the association between Sema5A and lymph node metastasis (LNM) in cervical cancer has been linked to three mechanisms. First, Sema5A induces lymphangiogenesis and the contact between tumor cells and lymphatic endothelial cells. Second, Sema5A induces MMP-2 and MMP-9 that mediate the invasive behavior of cervical cancer cells, which involves PlexinB3 receptor and the intracellular PI3K/AKT pathway. Finally, Sema5A/PlexinB3 signaling promotes lymphangiogenesis by inducing Met-mediated VEGF-C expression. Therefore, Sema5A is considered a prognostic indicator in cervical cancer 58.

**Endometrial cancer.** Endometrial cancer is the sixth most common cancer in women worldwide. In endometrial carcinoma, lymph node invasion is critical for choosing the treatment. The transcriptome of endometrial cancer samples was investigated with the aim of predicting the risk for lymph node metastatic invasion (LN + /LN- status) prior to lymphadenectomy. Interestingly, one of the identified biomarkers of LN- status is **Sema3D** 59, interacting with neuropilins and plexin receptors. Sema3D suppressor activity in this context may be explained by the functional competition with lymphoangiogenesis-inducing factors VEGFC and VEGFD, which have also been reported to bind neuropilins.

**Esophageal-Gastric cancer**. The esophageal carcinoma is the eighth most common cancer worldwide and it is divided into two major histological types: esophageal squamous cell carcinomas and esophageal adenocarcinomas. Gastric cancer is another major cause of death worldwide; notably, chronic infection by *Helicobacter pylori* is causative of gastric cancer. Gastric cardia adenocarcinoma was formerly associated with esophageal or gastric carcinomas; however, due to the distinct epidemiological and biological characteristics, these tumors are now diagnosed independently.

In esophageal squamous cell carcinoma (ESCC) a 3p chromosomal deletion is one of the most common genetic changes. Notably, chromosome 3 allelic loss and promoter hypermethylation are considered the main causes of **Sema3B** downregulation, and Sema3B reduced expresson was detected at mRNA and protein levels in ESCC tumor specimens 60. Furthermore, the downregulation of Sema3B was significantly associated with lymph node metastasis, advanced clinical stage and poor patient survival. A multivariate analysis indicated that downregulation of Sema3B could be used as an independent prognostic predictor for ESCC patients. It is well known that Sema3B is involved in the regulation of apoptosis and cell growth in different tumor types; indeed Sema3B can block ESCC cells at the G1/S checkpoint through inhibition of the PI3K/AKT signaling transduction pathway, upregulation of p53 and p21 expression and downregulation of cyclin D1 expression. Therefore Sema3B may be an important tumor-suppressor gene lost during the malignant progression of ESCC, and a valuable prognostic marker for ESCC patients 60. Also in gastric cardia adenocarcinoma (GCA) the loss of heterozygosity in chromosome 3p is one of the most frequent events, likely to impact on the expression of putative tumor suppressor genes, including *SEMA3B*. Notably, miR-6872 was identified within an intron of *SEMA3B* gene 61; notably, intronic miRNAs are co-expressed and may play synergetic role with their host genes. Furthermore, promoter hypermethylation impacted on the expression of SEMA3B-AS1, a long non-coding RNA (lncRNA) encoded by the complementary genomic DNA strand. In 2019, Guo et al., reported a similar expression trend of SEMA3B, SEMA3B-AS1, and miR-6872-5p in GCA tissues, as well as a common activity suppressing gastric cancer cell proliferation, migration, and invasion. Antisense lncRNAs can regulate the expression of sense protein coding genes in* cis*, and in this study SEMA3B-AS1 was found to induce SEMA3B expression. Downregulation of miR-6872-5p has been detected in GCA tissues and gastric cancer cells, and it may act as tumor-suppressor miRNA synergistic with its host gene SEMA3B, inhibiting GCA cell migration and invasion. In summary, SEMA3B, SEMA3B-AS1, and miR-6872-5p seem to play a suppressor role in GCA tumor development, and their expression levels are coregulated by aberrant promoter hypermethylation and histone modification. Moreover, due to the association of SEMA3B, SEMA3B-AS1, and miR-6872-5p expression with cancer prognosis, they represent potential prognostic markers for predicting GCA patient survival 61.

In a preliminary study, the expression of **Sema6B** in GCA primary samples was positively correlated with the presence of metastasis; moreover, Sema6B-silencing in a gastric tumor cell line suppressed migration and invasion, suggesting a potential relevance of this gene as GCA promoter 62.

The tumor microenvironment plays a major role in gastric carcinoma progression. In particular, this is characterized by a chronic inflammatory scenario comprising, among other cells, tumor-associated macrophages (TAMs). In 2018, Li et al. demonstrated that **Sema4D** is highly expressed in gastric carcinoma tissues, and its levels were significantly associated with the histological subtype, TMN stage, and lymph node metastasis. Notably, a significant correlation was also found with the distribution of the TAMs marker CD68, in gastric carcinoma tissues. This may indicate a functional association between Sema4D production and TAMs activity, which is an important factor promoting invasion and metastasis. Moreover, the combined detection of CD68 and Sema4D protein markers was shown to have a potential for predicting gastric carcinoma progression and prospective patient prognosis 63.

**Colorectal cancer**. Colorectal cancer (CRC) is considered a prime example of step-wise carcinogenesis, since the number of genetic alterations increases with the advancing histopathological stage of the disease, from early adenomatous lesions to invasive carcinomas and metastatic cancers. Recent studies revealed a close correlation between **Sema3D** levels and CRC disease progression. In fact, gene expression profiling demonstrated that Sema3D levels are higher in normal colorectal mucosa than in CRC tissues 64. Consistent with data reported above for cervical cancer, the authors furthermore found that Sema3D expression in CRC was inversely correlated with lymph node metastasis; and Sema3D-silencing was found to promote cancer cell migration *in vitro,* consistent with the idea of a metastasis-inhibitory activity of this semaphorin. Univariate survival analysis of CRC patients showed that tumors expressing high levels of Sema3D are associated with longer survival than those expressing low levels. Moreover, secreted Sema3D can be detected into patients' blood, and it may be considered a potential serological marker of CRC progression. Indeed, Sema3D serum levels were found to be significantly reduced in CRC patients compared with normal healthy controls. Taken together, these findings indicate that Sema3D may function as a tumor suppressor gene during the formation and development of CRC and it might also represent a biomarker for the early diagnosis and prognostic assessment of CRC 64. The implicated receptors and Sema3D signaling mechanisms were unfortunately not investigated in this work.

Multivariate Cox regression analyses revealed that **Sema4C** expression is an independent prognostic predictor of shorter overall survival of CRC patients. Sema4C levels in tumors were correlated with an EMT gene expression profile and its expression was enriched in CRC bearing a consensus molecular subtype 4 (CMS4) signature 65.

As for other cancers, **Sema4D** expression has furthermore been associated with CRC development. In particular, one study correlated Sema4D levels with those of hypoxia-inducible factor-1α (HIF-1α), suggesting that it is more abundantly expressed in hypoxic tumors. An elevated expression of Sema4D was closely associated to the presence of lymphatic metastasis, and to poorly differentiated tumors with advanced tumor stage. Multivariate Cox analysis revealed that Sema4D levels represent an independent indicator of poor prognosis in CRC patients 66. These findings are consistent with those of a subsequent study, which further revealed that the expression of Sema4D-receptor PlexinB1 in CRC samples correlated with lymph node metastasis; moreover, the combined elevated expression of both Sema4D and PlexinB1 was found to be a stronger independent risk factor for disease relapse, in a multivariate analysis 67.

Interestingly, Sema4D might also represent a potential therapeutic target for CRC patients. In fact, anti-angiogenic VEGF-targeted drugs are currently applied in combination with chemotherapy for CRC treatment. Emerging evidence has shown that Sema4D may represent an alternative pro-angiogenic factor produced by tumor cells in response to anti-VEGF therapy, responsible for resistance to therapy. Indeed, anti-angiogenic drugs upregulated Sema4D levels in cancer cells and tumor tissues, and Sema4D can exert significant proangiogenic activity in tumors 68. Thus, based on these data, targeting Sema4D could represent a treatment option for cancer patients developing adaptive resistance to anti-VEGF drugs. Such an approach is now amenable due to the development of Sema4D-blocking antibodies assayed in clinical studies 46; however, experimental evidence *in vivo* about the effect of combined treatments is currently missing.

**Pancreatic cancer**. Pancreatic ductal adenocarcinoma (PDAC) is the most prevalent form of pancreatic cancer and also the most lethal of all common cancers. This is largely due to its indolent growth in early stages and late diagnosis when already locally invasive, metastatic and resistant to most conventional chemotherapeutics. Genomic studies uncovered genetically altered molecular pathways in PDAC that may regulate the metastatic process; in particular, genes encoding semaphorins and their receptors were found to be among the cellular pathways most frequently altered at the genetic level 69,70. Notably, immunohistochemical detection of high **Sema3D** expression in primary PDAC was associated with wide metastatic disease and decreased patient survival. Moreover, Sema3D-depeleted cancer showed decreased invasive and metastatic potential in culture and in mouse models. Thus, Sema3D may represent a novel therapeutic target and prognostic biomarker of metastatic pancreatic cancer 71.

The expression of** Sema5A** was found to be significantly higher in PDAC samples compared to paired normal adjacent tissue derived from the same patient; moreover, Sema5A levels were further increased in metastatic lesions 72. In this study, Sema5A expression increase was similarly associated with the progression of neuroendocrine pancreatic tumors (PNET). *In vitro* assays indicated that Sema5A is actively stimulating pancreatic cancer cell migration, via PlexinB3 receptor-associated Met tyrosine kinase; however gene knock-down experiments revealed that the role of Sema5A in PDAC is complex and needs further elucidation 73.

The study of mouse PNET models revealed that **Sema3A** expression is commonly downregulated in tumors, compared to normal tissues, consistent with the known role of this semaphorin as angiogenesis inhibitor. Moreover, the localized delivery of Sema3A effectively tamed tumor angiogenesis, leading to vessel normalization and to a significant inhibition of hypoxia-driven metastatic spreading 54. In another study based on IHC analysis, Sema3A expression with its co-receptor NRP1 in PDAC was instead correlated with poor patient prognosis 74. In order to avoid any undesirable NRP1-mediated activities of Sema3A (e.g. see 75), an engineered Sema3A-derived recombinant molecule was developed for systemic delivery, which selectively elicited plexin-dependent therapeutic effects in both PDAC and PNET mouse models 55. Considering the broad relevance of Sema3A activity in various tumor types, this molecule could represent a promising tool for cancer treatment.

**Head and Neck Cancer**. Head and neck squamous cell carcinoma (HNSCC) arises from epithelial cells found in the mucosa of the oral cavity, lips, larynx, pharynx, and nasal passages. It is a locally aggressive neoplasm, invading the adjacent bones of the face, with propensity to early metastatic dissemination.

**Sema3A** expression was an independent prognostic predictor of overall survival of HNSCC patients, correlating lower expression with poor outcome. This putative tumor suppressor activity was confirmed by experimental studies in cultured cancer cells and in preclinical mouse models 76.

In contrast, high **Sema4D** expression in HNSCC was significantly associated with bone invasion 77. Moreover, consistent with its activity promoting osteoclastogenesis and bone resorptive activity, the knock-down of Sema4D in HNSCC cells reduced bone invasion in preclinical mouse models. Sema4D secreted by cancer cells was also found to regulate the tumor microenvironment, promoting the recruitment from the bone marrow of myeloid-derived suppressor cells 78, and the formation of a dense fibrotic peri-tumoral stroma 79, both hindering anti-cancer immune response. Notably, Sema4D was detected in the plasma of head and neck cancer patients at significantly higher levels compared to healthy donors 79.

**Lung cancer**. For several decades, largely linked to tobacco smoke, lung cancer has been the most common cancer in the world. Now, it remains the third most common cancer, but it is well recognized as heterogeneous disease, comprising several different subtypes. Based on main histotype, prognostic, and therapeutic implications, lung cancers are divided in two main groups: small-cell carcinoma (SCLC) and non-small-cell carcinoma (NSCLC). NSCLCs have the highest incidence, and are generally insensitive to chemotherapy and radiation therapy. They essentially consist in three subtypes: squamous cell carcinoma, large cell carcinoma, and adenocarcinoma; although all lung cancer subtypes are strongly associated with exposure to tobacco smoking, adenocarcinoma is the most common subtype in never-smoker patients.

As mentioned above,** SEMA3B** is located in a site of frequent allele loss and hypermethylation in lung cancers, and experimental evidence indicated its role as tumor suppressor gene also in this context 8,80,81. In experimental preclinical models in mice, the forced expression of Sema3B impaired lung cancer growth, possibly accounted by anti-angiogenic activity.

**Sema4B** has also been recently implicated as a suppressor of NSCLC tumorigenesis, due to its ability to inhibit the PI3K pathway and thereby activate FoxO1 transcriptional factor, negatively controlling cancer cell proliferation. Experimental evidence *in vivo*, in mouse models, indicated that Sema4B expression in NSCLC is a critical determinant of tumor progression, and suggested its relevance as novel therapeutic target in this tumor type 82.

One of the issues of therapy resistance in NSCLC is due to a peculiar mechanism named vasculogenic mimicry (VM), based on vascular tubes formation by tumor cells undergoing plastic reprogramming to acquire an endothelial-like phenotype. Notably, it was observed an increased expression on **Sema4D** in NSCLC tumor samples compared to adjacent normal tissues, and Xia et al. demonstrated, *in vitro* and in mouse models, that Sema4D-overexpressing NSCLC tissues are prone to form VM structures 83. According to prior reports, the Sema4D receptor PlexinB1 controls the RhoA/ROCK pathway, which is also involved in VM formation. Therefore, Sema4D may represent a useful biomarker predicting tumors likely to develop VM channels and become resistant to anti-angiogenic therapies; moreover, the availability of Sema4D-blocking antibodies could potentially allow to target this semaphorin in combined therapeutic regimens.

**Sema5A** downregulation in lung adenocarcinoma tissues was associated with poor overall survival. Furthermore, since transmembrane Sema5A can be cleaved by ADAM17 metalloproteases, this mechanism contributes to the downregulation of surface‑exposed Sema5A in lung adenocarcinoma cells 84. *In vitro* and *in vivo* experiments with NSCLC cellular models indicated a clear tumor suppressor activity of Sema5A. These findings suggested a suppressor role for Sema5A in lung adenocarcinomas and a potential relevance as prognostic biomarker 84.

A recent work demonstrated that low** Sema6A** expression correlates with high tumor recurrence rates in lung cancer patients 85. Moreover, the authors found that Sema6A could suppress NSCLC cell migration *in vitro*, by the activation of NRF2/HMOX1 axis. NRF2 is considered a tumor suppressor, and HMOX1 its downstream effector to suppress lung cancer cells migration, via the transcriptional regulation of genes such as plasminogen activator urokinase (PLAU), MMP1, MMP9, and IGF binding protein-3 (IGFBP3). These data indicated that Sema6A acts as a cancer suppressor and a potential biomarker of less aggressive lung cancers 85.

The onset of mutations causing resistance to EGFR tyrosine kinase inhibitors (EGFR-TKIs) represents a clinical challenge for the treatment of lung cancer. Notably, Kinehara and colleagues found that **Sema7A** is highly induced by the EGFR pathway, via mTOR kinase activity. Moreover, since mTOR represent an effector hub for various external stimuli, Sema7A expression can be induced by additional oncogenic upstream signals beside EGFR. Indeed, the combined treatment of EGFR-mutated cells with an mTOR inhibitor resulted in higher sensitivity to therapy with EGFR-TKIs. Based on this study, Sema7A levels may be considered a biomarker of mTOR pathway activation, useful for guiding therapeutic decisions in EGFR-dependent lung adenocarcinoma patients. In addition, experimental data indicated that Sema7A-Integrin-beta1 signaling itself promotes ERK activation, which can bypass the impact of EGFR-TKIs, featuring a candidate therapeutic target as well as a putative novel biomarker for predicting drug-resistance in patients with EGFR-mutated lung adenocarcinoma 86.

**Bladder cancer.** Bladder cancer (BC) is a poorly treatable chronic disease, with a high tendency to recur and progress. Ideally, the assessment of relevant biomarkers in urine samples could be a convenient method for early BC detection*.* It was recently reported that** Sema3A** levels in urine are significantly correlated with presence of urothelial cancer 87. Consistently, histopathological analyses proved that the expression of Sema3A in bladder tissue increases with the tumor grade. Based on these data, a combined analysis of urine Sema3A levels and urine sediment cytology could represent a significant biomarker for early BC diagnosis, with relatively high sensitivity and specificity.

**Prostate cancer.** Prostate cancer (PC) is possibly the most frequent tumor in males, although it often remains at subclinical level. Despite the compliance of early-stage PC to surgery and radiation therapy, locally advanced and metastatic prostate carcinomas remain a clinical challenge. Normal prostate and carcinoma cells typically express the androgen receptor (AR) and are dependent on its signaling. Thus, chemical castration is often used as a first-line therapy for advanced disease; however, the onset of hormonal therapy resistance is usually ensuing, largely through mutations of the androgen receptor (AR), aberrant AR signaling, or AR bypass mechanisms. Interestingly, it was found that AR activity induces **Sema3C** expression, via the GATA2 transcription factor 88. Actually, Sema3C is a secreted semaphorin with a pleiotropic and partly controversial activity in the tumor microenvironment, as it was reported to variedly regulate epithelial-to-mesenchymal transition, cell migration angiogenesis, and cancer cell stemness 89. This could be explained by the fact that Sema3C may trans-activate multiple receptor tyrosine kinases involved in the growth of prostate carcinoma cells, and potentially also implicated in resistance to androgen-deprivation therapies 90. In fact, Lee et al. identified several small molecule inhibitors of Sema3C that reduce the phosphorylation of EGFR, HER2/ErbB2, SHC, and MAPK, collectively providing evidence in experimental models that Sema3C inhibition may represent a promising avenue for developing targeted therapies against PC and possibly other cancer types 91. Notably a recent bioinformatic analysis of class-3 semaphorin expression in human tumors (based on TCGA datasets) indicated a remarkable heterogeneity in the association of Sema3C levels with better or worse prognosis in diverse tumor types 50. Although these data may suffer from relevant discrepancy between gene expression at mRNA and protein levels, further analysis of Sema3C association with prostate cancer patients' prognosis and resistance to therapy is warranted.

The invasion of nerve sheaths by cancer cells, termed perineural invasion, is a key feature of prostate cancer; in particular, perineural invasion appears to be a major route for prostate cancer metastatic spreading, which is the main obstacle to cure. Interestingly, **Sema4F** expression in cancer cells has been correlated with the induction of axonal sprouting *in vitro*, and with increased nerve density and perineural invasion in PC 92. In addition, *in vitro* experiments indicated that Sema4F induces PC cell proliferation and migration. Notably, Sema4F expression in prostate cancer cells is independently predictive of tumor recurrence 92. Due to its activity to induce axonogenesis and perineural cancer cell invasion, Sema4F may also represent a potential target to inhibit PC progression. It is intriguing to note that, in an earlier study, Sema4F had been found to suppress the proliferation of differentiated Schwann cells ensheating axonal processes in peripheral nerves 93; this apparent discrepancy may suggest the involvement of different receptor complexes or signaling modes (forward or reverse), which are presently unknown.

**Hematological malignancies**. Multiple myeloma (MM) is a neoplastic disorder due to the proliferation of malignant plasma cells in the bone marrow, which is accompanied by upregulation of bone resorption, due to increased abundance and activity of osteoclasts, whereas bone formation is suppressed due to reduced osteoblast number and activity. MM is thought to evolve from a monoclonal gammopathy of undetermined significance (MGUS), which progresses to smoldering MM (SMM) and finally to MM.

**Sema3A** has an important role in bone homeostasis, mostly promoting bone formation. Bone-marrow-derived Sema3A can be found in serum in significant amounts; however, Lavi and colleagues found that its serum concentration drops significantly in patients with MM in advanced stage, but not in pre-neoplastic conditions, such as MGUS or SMM. The observed effect is consistent with the idea that the replacement of normal bone marrow with malignant cells depletes Sema3A production. It was thereby proposed that monitoring Sema3A levels could allow secondary prevention and early diagnosis, as well as serve as indicator of therapeutic response and prognostic marker of MM progression. Moreover, a promising tumor suppressor activity of Sema3A was also observed in this context, upon overexpression of its furin-cleavage resistant isoform in a mouse model of MM 94.

In a complementary scenario,** Sema4D** and its receptor, Plexin-B1, have been found elevated in the serum and bone marrow of patients with active MM 95. As discussed above for osteolytic metastasis, Sema4D is actually expressed by osteoclasts and negatively regulates osteoblast activity and bone formation 40. It therefore appears that MM is associated with decreased Sema3A-dependent bone formation, as well as upregulated Sema4D-dependent bone loss.

Sema3A and Sema4D were furthermore similarly implicated in leukemia cell regulation. In particular, **Sema3A** expression was found to be lower in leukemia cells compared to normal hematopoietic cells found in bone marrow 96 and Sema3A levels are significantly reduced in the serum of acute leukemia patients compared to healthy individuals 94. Consistent with the posited tumor suppressor role of this semaphorin, experimental studies *ex vivo* have shown that Sema3A can suppress the growth and promote the apoptosis of human leukemic cells, via diverse mechanisms; Sema4D, in contrast, was found to promote leukemia cell survival and growth 97.

**Gliomas.** Gliomas are the most prevalent primary tumors of the brain and spinal cord. They include a spectrum of subtypes, from localized grade-I tumors to infiltrating grade IV glioblastomas (GBM). **Sema3C** protein levels were analyzed in astrocytoma tissues of different malignancy grade, revealing a marked increase of expression in aggressive glioblastomas compared to lower grade astrocytomas (i.e. WHO grades I, II, and III) 98. Notably, Sema3C upregulation was distinctly associated with poor prognosis in glioma patients. These correlations were not confirmed based on analyses at mRNA level, although the significance of this discrepancy has not been explained.

GBM is the most aggressive glioma subtype, rapidly growing and highly invasive, making the median overall survival of GBM patients after diagnosis approximately only 12-15 months. In this context, low expression of **Sema6A** was associated with a larger tumor volume, as well as shorter time to recurrence and overall survival, featuring an independent prognostic factor for GBM patients 99. *In vitro* experiments showed that Sema6A could remarkably suppress the GBM cell migration, invasion, and viability. Thus, Sema6A might act as a candidate prognostic marker for GBM.

## Targeting semaphorin signaling for cancer therapy

As introduced above, a large body of evidence obtained in experimental models in culture and in mice demonstrated that semaphorins act as regulators of tumor progression, and that interfering with their functions can significantly alter disease outcome. These preclinical data have thus endorsed the idea of targeting semaphorin signaling pathways for the treatment of human cancers. Based on current knowledge, this could be aimed not only to hinder tumor progression, but also to foster responsiveness to standard and innovative therapeutic approaches, and curb mechanisms of drug resistance. An important issue to consider is the functional relevance of the targeted semaphorin signals in cancer versus normal tissues. In fact, although semaphorins are best known as developmental cues, they also regulate e.g. the immune function and bone homeostasis in the adult, which are potentially relevant fields both for cancer therapeutics and to check out any serious adverse effects. Notably, the functional role of these signals in the adult nervous system is poorly understood, but the potential impact of systemic-delivered semaphorin-targeted molecules may depend on their capacity to cross an integer blood-brain barrier.

In order to interfere with tumor-promoting semaphorins, function-blocking antibodies appear the most actual approach 100. Indeed, humanized anti-Sema4D antibodies are the first tool that has been successfully developed for clinical application. In particular, the safety and tolerability of VX15/2503 was validated in two independent phase-1 trials, enrolling over 50 patients bearing advanced solid tumors or multiple sclerosis (NCT01313065, NCT01764737). The drug was well tolerated, as treatment-related adverse events were mostly nausea, urinary tract infection, and fatigue (grade 1 or 2, around 10% prevalence each). Only one pancreatic cancer patient experienced a grade 3 dose-limiting liver toxicity 46 101. The mechanism of action of this Sema4D-targeted antibody is not fully understood, since this semaphorin was reported to act on cancer cells and many other cell populations in the tumor microenvironment 21. Consistent with its activity limiting CTL infiltration in tumors, this drug is currently under evaluation in three distinct Phase-1b/2 trials for the treatment of advanced non-small-cell lung cancers, head and neck squamous cell cancers, or pancreatic and colorectal cancers, in combination with the immune checkpoint inhibitors anti-PD-L1 avelumab (NCT03268057), or anti-CTLA-4 ipilimumab and anti-PD1 nivolumab (NCT03690986, NCT03373188), respectively. The development of further semaphorin-blocking molecules for clinical application is expected. Beyond antibodies, alternative approaches under consideration could be small molecules, which can ensure a better scalability of production and a wider organ biodistribution. However, so far, only the Sema3A inhibitors SM-216289/xanthofulvin and SM-345431/vinaxanthone were validated, which have been applied to promote tissue regeneration, e.g. in preclinical mouse models of spinal cord injury 102. Moreover, soluble extracellular domains of the plexins have been successfully used in experimental mouse models as decoy receptors capturing secreted semaphorin ligands, including for cancer treatment 14,90. It should be noted, however, that such approach may not be ideal for the functional blockade of transmembrane semaphorins, as it was found to trigger their reverse signaling in other studies 25 103.

Another potential option could be to directly target semaphorin receptors, e.g. by function-blocking antibodies. One such example, directed against PlexinB1, has been shown to inhibit breast cancer development in a mouse model 104. Notably, this approach could offer an opportunity to circumvent the functional redundancy between diverse semaphorins binding the same receptor; on the other hand, however, it is still unclear whether it may impinge on ligand-independent functions, warranting further investigation. This issue is particularly relevant in case of neuropilins (NRPs), which are promiscuous co-receptors for secreted semaphorins as well as for ligands of the VEGF family and other soluble growth factors 105,106. Moreover, NRPs can form signaling complexes with a variety of cell surface molecules, which enables them with a broad spectrum of biological activities, also in cancer context. Intriguingly, while NRPs can bind many tumor-suppressor semaphorins, they are in fact considered tumor-promoting molecules per se; thus, NRP1 targeting is gaining increasing interest for cancer therapy, independent of semaphorin functions 107,108.

On a complementary line of intervention, semaphorins that are often downregulated in human cancers, and have been found to suppress tumor development in preclinical models, could in principle be used as a blueprint to design novel anti-cancer drugs. In fact, the systemic administration of two engineered semaphorin molecules, including a NRP1-independent Sema3A superagonist, has been assayed in mice, achieving successful inhibition of tumor growth and metastatic dissemination, without significant adverse effects 16,55. Notably, the development of large recombinant proteins applicable for clinical application is still a challenge, and first-in-men trials with this kind of semaphorin superagonists are hopefully forthcoming. Cross-linking antibodies or other molecular tools capable of achieving ligand-independent activation of semaphorin receptors could represent another avenue for investigation.

## Conclusions

Semaphorin signals have a multifaceted role in tumor development, which is still under deep investigation. They can regulate cancer cell behavior, but also control tumor angiogenesis and the function of immune cells recruited from the circulation, among other things. Interestingly, Sema4F-driven perineural invasion of prostate cancer cells, associated with tumor progression, highlighted another potentially important aspect of their activity in this context, deserving further studies.

Accumulating evidence indicates that selected semaphorins may represent useful biomarkers for the diagnosis and especially the prognostic evaluation of various human tumors. For instance, the elevated expression of certain family members has been recurrently associated with advanced tumor stage and poor patient prognosis. Certain semaphorins are also promising predictors of the response to anti-cancer therapies, including standard chemo/radiotherapy (e.g. Sema3C, Sema4C), anti-angiogenic drugs (e.g. Sema4D), anti-hormonal treatments (e.g. Sema4C, Sema7A), and other targeted molecular therapies, such as with anti-EGFR inhibitors (Sema7A). Moreover, preclinical evidence supported the identification of some of these molecules as relevant targets for therapy themselves. For instance, antibodies directed against Sema4D, which is a prominent example of this kind, are currently under assessment in clinical trials. It should be noted that semaphorin activity in the tumor microenvironment may be complicated by potential diverse impacts in target cells characterized by distinctive receptor complexes, which warrants further investigation of the anti-tumor mechanisms of action of these drugs.

On the other hand, semaphorins that are considered putative tumor suppressors based on experimental evidence, are frequently downregulated in human cancer cells compared to normal counterparts, and their expression may represent a biomarker of better patient prognosis. Actually, the mechanisms of semaphorin disregulation in tumors are still largely unknown. Mutations have been infrequently reported, while genomic hypermethylation seems to be responsible for the downregulation of semaphorins acting as tumor suppressors. Novel regulatory mechanisms are also emerging, such as for example the expression of intronic miRNAs and long non-coding RNAs (lncRNAs) associated with Sema3B. In some cases (e.g. Sema3A), the systemic delivery of these “inhibitory” semaphorins for cancer therapy has been validated in preclinical studies resulting in inhibition of tumor progression. Thus, a better understanding of the implicated tumor-suppressive mechanisms will enable an optimized targeting of these molecules, and the identification of predictive markers of responsiveness to prospective “semaphorin-based” therapies.

## Figures and Tables

**Table 1:** Putative clinical relevance of disregulated expression of semaphorins in human tumors

SEMAPHORIN	TUMOR TYPE	RESULTS	REFs
**Sema3A**	Breast cancer	- High levels in tumors correlate with better patient prognosis.	12
	Pancreatic cancer	- Putative suppressor (downregulated in tumors). - Coexpression with NRP1 correlated with poor patient prognosis - Therapeutic effect in preclinical models of Sema3A-derived NRP1-independent recombinant molecule	54,55,74
	Head and neck cancer (HNSCC)	- High expression as independent prognostic predictor of better patient survival; tumor suppressor activity in preclinical studies	76
	Bladder cancer	- Overexpression in tumors of high grade. - Detection in urine is a cancer biomarker	87
	Multiple myeloma and acute leukemias	- Serum concentration drops in patients with advanced bone marrow diffusion; tumor suppressor activity.	94
	Ovarian cancer	- Low levels correlate with higher FIGO stage, histological grade, lymphatic metastasis and distant metastasis.	53
			
**Sema3B**	Breast cancer	- Downregulated expression; and tumor suppressing/anti-metastatic activity in preclinical models.	9
	Lung cancer	- Downregulated expression; and tumor suppressing/anti-metastatic activity in preclinical models.	8,80,81
	Esophageal squamous cell carcinoma	- Low expression associated with lymph node metastasis, advanced clinical stage and poor patient survival; tumor suppressor activity.	60
	Gastric cardia adenocarcinoma	- Suppressor role in tumor development.	61
			
**Sema3C**	Glioblastoma	- Marked increase of expression in aggressive tumors.	98
	Ovarian cancer	- Experimental evidence of its role in cancer cell resistance to cisplatin treatment. Consistently, a broad bioinformatic analysis indicated a significant inverse correlation between Sema3C expression and sensitivity to anti-cancer drugs.	49,50
			
**Sema3D**	Endometrial cancer	- Biomarker of low risk of lymph node metastatic dissemination.	59
	Colorectal cancer	- Tumor expression inversely correlated with lymph node metastasis. - Serum levels reduced in CRC patients compared with normal healthy controls.	64
	Pancreatic cancer	- High tumor expression associated with metastatic disease and poor patient survival	71
			
**Sema3E**	Breast cancer	- Higher expression in tumors associated with distant metastasis. - A recombinant modified isoform suppresses tumor growth and metastasis in preclinical models.	14 16
	Ovarian cancer	- Elevated expression has been associated with high-grade ovarian endometrioid carcinomas	52
			
**Sema4C**	Breast cancer	- High levels (in tumor and serum) correlate with metastasis and poor patient survival. - Sustains resistance to paclitaxel-based chemotherapy in cancer cells. - Driver of metastatic progression in experimental models.	23,24,25, 26,27
	Cervical cancer	- Sustains resistance to cisplatin-based chemotherapy in cancer cells.	56
	Colorectal cancer	- Independent prognostic predictor of shorter survival. - Higher levels in consensus molecular subtype 4 (CMS4)	65
			
**Sema4D**	Breast cancer	- Higher expression in estrogen-positive tumors, associated with better prognosis. - Reduced expression in patients treated with tamoxifen. - Higher expression in primary tumor is associated with brain metastasis formation. - anti-Sema4D antibodies enhanced the efficacy of immune checkpoint inhibition in preclinical tumor models.	38,41,42 44,45
	Ovarian cancer	- High levels associated with high malignancy.	48
	Cervical cancer	- High expression associated with lymph node metastases and unfavorable outcome.	57
	Gastric carcinoma	- High expression associated with lymph node metastasis.	63
	Colorectal cancer	- Elevated expression correlated with lymph node metastasis and it was an independent indicator of poor prognosis. - High expression is a risk factor for disease relapse. - Potential therapeutic target in patients developing adaptive resistance to anti-VEGF drugs.	66,67,68
	Head and neck cancer (HNSCC)	- High expression is associated with infiltration of myeloid-derived suppressor cells, presence peritumoral fibrotic stroma, and metastatic bone invasion. - Plasma levels higher in patients compared to healthy individuals.	78,79,77
	Multiple myeloma	- Elevated levels in serum and bone marrow.	95
			
**Sema4F**	Prostate cancer	- High expression correlates with increased nerve density and perineural invasion. - Predictive biomarker of tumor recurrence	92
			
**Sema5A**	Cervical cancer	- High tumor expression associated with lymphangiogenesis, metastatic progression and poor patient prognosis.	58
	Lung cancer (NSCLC)	- Low expression associated with poor patient survival; putative tumor suppressor.	84
			
**Sema6A**	Lung cancer (NSCLC)	- Low expression correlate with high tumor recurrence rates; tumor suppressor activity.	85
	Glioblastoma	- Low expression associated with larger tumors, shorter time to recurrence and overall survival; tumor suppressor activity.	99
			
**Sema6B**	Gastric cardia adenocarcinoma	- High expression in tumor correlated with the presence of metastasis; putative promoter activity.	62
			
**Sema7A**	Breast cancer	- Expression in tumors is associated with shorter patient survival, disease recurrence, and resistance to hormonal therapy. - Evidence of tumor promoter role in preclinical models, conferring resistance to estrogen deprivation and promoting disease progression and metastasis.	28,29,30 31,32
	Lung cancer (NSCLC)	- Biomarker of mTOR pathway activation, and predicting drug-resistance in EGFR-mutated tumors.	86

## Abbreviations

AI: aromatase inhibitors
AR: androgen receptor
BC: bladder cancer
CRC: colorectal cancer
EGFR: epidermal growth factor receptor
EGFR-TKIs: EGFR tyrosine kinase inhibitors
EMT: epithelial mesenchymal transition
ER: receptor for estrogen
ESCC: esophageal squamous cell carcinoma
GBM: glioblastoma
GCA: cardia adenocarcinoma
HER2: human epidermal growth factor receptor 2
HIF-1α: hypoxia-inducible factor-1α
HMOX1: Heme-oxygenase-1
HNSCC: head and neck squamous cell carcinoma
HPV: human papillomavirus
IGFBP3: IGF binding protein-3
IHC: immunohistochemistry
LECs: lymphatic endothelial cells
lncRNA: long non-coding RNA
LNM: lymph node metastasis
MGUS: monoclonal gammopathy of undetermined significance
MM: multiple myeloma
MMPs: matrix metalloproteinases
NRF2: Nuclear Factor, Erythroid-2-Like-2
NRP: neuropilin
NSCLC: non-small cell lung carcinoma
PC: prostate cancer
PDAC: pancreatic ductal adenocarcinoma
PI3K: phosphatidylinositol-3-kinase
PLAU: plasminogen activator urokinase
PNET: neuroendocrine pancreatic tumors
PR: receptor for progesterone
PSI: plexin-semaphorin-integrin
SCLC: small cell lung carcinoma
Sema: sempahorin
SMM: smoldering multiple myeloma
TAMs: tumor associated macrophages
VEGF: vascular endothelial growth factor
VM: vasculogenic mimicry
